# Congenital anomalies among newborn babies in Felege-Hiwot Comprehensive Specialized Referral Hospital, Bahir Dar, Ethiopia

**DOI:** 10.1038/s41598-021-90387-0

**Published:** 2021-05-26

**Authors:** Daniel Mekonnen, MollaTaye, Walelegn Worku

**Affiliations:** 1 Department of Public Health, Haramaya University, Dire Dawa, Ethiopia; 2 Department of Anatomy, School of Medicine, College of Medicine and Health Sciences, The University of Gondar, Central Gondar, Ethiopia; 3 Department of Occupational and Environmental Health, Institute of Public Health, College of Medicine and Health Sciences, The University of Gondar, Central Gondar, Ethiopia

**Keywords:** Anatomy, Medical research

## Abstract

Congenital anomaly is a structural or functional defect which could occur in any organ system. The aim of this study was to determine the prevalence and associated factors of congenital anomalies among newborn babies delivered at Felege-Hiwot Comprehensive Specialized Referral Hospital, Bahir Dar, Ethiopia. A cross-sectional study design was used to review medical records/charts of 11,177 new born babies born at the delivery ward of Felege-Hiwot Comprehensive Specialized Referral Hospital, Ethiopia. The data were collected using an abstraction form. A bivariate analysis was done to assess factors associated with congenital anomalies. Variables whose p-value < 0.2 were included in the multivariable analysis to identify the effects of confounders. P-values < 0.05 were considered statistically significant. A total of 11,177 newborn babies and their mothers were included in the study and the proportion of congenital anomalies was found to be 0.62%. The most (46.4%) prevalent congenital anomaly was neural tube defects. Newborn birth weight < 1.25 kg [AOR, 32.6, 95% CI 11.9–89.0], and newborn weight < 2.5 kg [AOR, 2.67, 95% CI 1.54–4.65], antenatal visits [AOR, 4.0, 95% CI 2.39–6.69] and urban residence [AOR, 2.1, 95% CI 1.28–3.55] were statistically significant factors. In conclusion, neural tube defects were anomalies prevalent in this study. Antenatal visits, birth weight and residence were factors associated with congenital anomalies.

## Introduction

Congenital anomalies (CAs) also known as birth defects are any body organ system structural and functional abnormality, which may be detected during pregnancy, delivery or later in life^1,2^. A congenital anomaly (CA) causes neonatal and infant morbidity and mortality. The World Health Organization (WHO) estimated that in 2008 about 260,000 babies born with birth defect die during the first 28 days of life^3^.

Globally, about one in every 33 babies are born with a birth defect^4,5^. It is also estimated that 3–7% of children are born with birth defects annually^6^. The prevalence of CAs varies from one country to another and even among regions of the same country^7–9^. A study in the United States of America and the United Kingdom showed the prevalence of CAs was 6% and 3.3%, respectively^10^. In addition, the prevalence of CAs varies among communities which do the same jobs and have similar living styles and areas^1,6,7^. Moreover, the problems have a significant socio-economical and psychological impact on individuals, families and communities^6^.

The exact etiologies of many of the CAs are unknown although the causes of a few CAs are known to be related to multifactorial origins and genetic^7,11–14^ and environmental factors^12,15–17^. Likewise, consanguineous marriages and chromosomal defects like nervous system problems were reported as causes of CAs^18,19^. However, such marriages are not common among Ethiopian women due to social and cultural norms.

In addition, a family history of birth defects has been associated with an increased risk of having another child with congenital anomalies, with a recurrence rate ranging between 2 and 5% for neural tube defects and Down syndrome, respectively^20^. Similarly, Fouzia et al., (2013) reported that neural tube defects were more prevalent among families in low socioeconomic and educational status^21^.

Births of children with anomaly are stressful situations for parents as well as the community. In Ethiopia, many children die and becoming disabled by CAs. Furthermore, when mothers have babies with CAs, communities attribute it to sin or God’s anger; as a result, parents are likely to feel anxious and guilty. However, the problem is not given due attention by policy makers and researchers. The aim of this study was to assess the proportion and associated factors of CAs among babies newborn at Felege-Hiwot Comprehensive Specialized Referral hospital, Bahir Dar, Ethiopia.

## Methods

### Study area

The study was conducted in Bahir Dar, the capital of the Amhara Regional state, 560 km from Addis Ababa, the capital of Ethiopia. The city is bounded by the Blue Nile River and Lake Tana. The city is divided into seven sub cities and has seven health centers and two hospitals (one public and one private). According to the 2011 census^22^, the city had a total population of 273,000, the majority of whom were Amhara. In 2002, the city was awarded the UNESCO Cities for Peace Prize for addressing the challenges of rapid urbanization. Felege-Hiwot Comprehensive Specialized Referral, a tertiary level hospital, provides various services including delivery. The hospital provides a large number of deliveries using its patient diagnosing devices, such as ultrasound, x-ray, electrocardiography machine, Medical examination by using computer to produce inside body image (CT scan) by radiologists, pediatricians and other experienced medical doctors who can diagnose CAs.

Chat chewing seemed the most common practice in Bahir Dar. According to the 2011 Ethiopia Demographic and Health Survey, the prevalence of alcohol consumption was (78%) among women in the region, which is secondary to Tigray region (86%)^22^.

### Study design

A retrospective cross-sectional study design was used to assess the quantitative proportion of a CA and their associated factors.

### Study and source population

The population and participants of the study were all newborn babies who were delivered at Felege-Hiwot hospital from Sept. 2009 to Aug. 2014. The inclusion criteria was all new born Ethiopian babies and the exclusion criteria was babies who are not Ethiopian and who are not delivered at Felege-Hiwot Comprehensive Specialized Referral Hospital.

### Sample size and method

All new born babies delivered in Felge-Hiwot Comprehensive Specialized Referral hospital from Sept. 2009 to Aug. 2014 were included in the study. Medical records/charts were reviewed to identify new born babies with CAs.

### Data collection procedures and techniques

Data were collected from hospital records/charts using data abstraction forms. The charts of the mothers of the newborns were reviewed carefully before the required data were collected by the primary investigator and trained nurses/midwives. The principal investigator supervised the process daily and excluded incomplete data.

### Data processing and analysis

The data were checked for completeness and records without main variables were considered as incomplete and rejected. The data were entered into Epi-info 3.5.1 software and transferred to SPSS version 20 for analysis. A bivariate analysis was carried out to assess the statistical significance between the independent and outcome variables by using a 95% CI and the Odds Ratio (OR) to determine the variables which could be included in the multivariable analysis. All variables with a p-value < 0.2 in the bivariate analysis were included in the multivariable logistic regression analysis. All significant variables in the bivariate variables were computed together to prevent confounders. P-values < 0.05 were taken as a statistically significant factor for CAs.

### Data quality control

The data were collected carefully by the principal investigator and nurses/midwives who were trained for 2 days prior to data collection. A strict process supervision and daily data checkups were carried out by the principal investigator. Incomplete data were filled again by data collectors; however, data which were not recorded (missed) on charts/recording logbooks by health workers were rejected.

### Ethical considerations

Ethical clearance and waiver were obtained from the Institutional Review Board of Addis Continental Institute of Public Health and Haramaya University joint master of public health program ethical clearance committee and was submitted to medical director/manager of Felege-Hiwot Comprehensive Specialized Referral hospital. Finally, the hospital manager/medical director permitted the investigators to use the data. Since the study was conducted by reviewing charts/medical records, waiver and informed consent was obtained from the hospital manager/medical director to collect the required information from the study subject’s charts who didn’t present in the hospital during the data collection period. In addition, informed consent was also obtained from baby’s mothers who were present in the hospital for medical service at the time of data collection. Furthermore, the study was carried out according to national and international ethical guidelines.

After obtaining permission from the hospital manager/medical director, relevant data were gathered from the hospital records/charts. Information taken from the study subjects’ charts were kept anonymous by omitting their names and other identifications. All of the information was kept confidential, only the researchers and data collectors had access to it.

### Ethical approval and consent to participate

This study granted ethical approval from Addis Continental Institute of Public Health and Haramaya University joint MPH program ethical clearance committee. The hospital manager and medical director gave permission.

## Results

A total of 11,177 newborn babies delivered at Felege-Hiwot Comprehensive Specialized Referral hospital from Sept. 2009 to Aug. 2014 and their mothers were included in the study. Out of these, 69 babies were identified with congenital anomalies. The overall proportion of congenital anomalies were 0.62%. Of the total 69 babies with CAs, 52% were male. The number of newborn babies and their mothers included in each variable varied based on the completeness of the data documented in the charts/recording logbooks.

### Socio-demographic characteristics

The majority (71.9%) of the babies mothers were between 25 and 34 years old, with the mean age of 28.08 years and standard deviation of 4.59. Almost three fourths (73.5%) of the mothers lived in rural areas and about 47.2% of them were farmers (Table 1).

**Table 1:** Socio-demographic characteristics of newborn baby’s mothers delivered at Felege-Hiwot Comprehensive Specialized Referral hospital, from Sept. 2009 to Aug. 2014, Bahir Dar, Ethiopia.

Characteristics	Frequency (%)
**Mother age(n = 11,177)**	
15–24 years	2209 (19.8%)
25–34 years	8041 (71.9%)
≥ 35 years	927 (8.3%)
**Residence (n = 11,177)**	
Urban	2965 (26.5%)
Rural	8212 (73.5%)
**Marital status (n = 893)**	
Never married	12 (1.3%)
Married	880 (98.7%)
**Mother education (n = 628)**	
No formal education	371 (59.1%)
Formal education	257 (40.9%)
**Mother occupation (n = 1587)**	
Farmer	749 (47.2%)
Office work	280 (17.6%)
House wife	558 (35.2%)

### Maternal characteristics

Out of the 11,177 mothers, 96 (0.9%) were known diabetic and 1207 (10.8%) delivered by caesarean section (CS); 2647 (23.7%) had one and more antenatal (ANC) visits; out of 1342 of the mothers, 1339 (99.8%) took multivitamins (Table 2).

**Table 2:** Reproductive characteristics of newborn baby’s mothers delivered at Felege-Hiwot Comprehensive Specialized Referral hospital, from Sept. 2009 to Aug. 2014, Bahir Dar, Ethiopia.

Characteristics	Frequency (%)
**Parity (n = 11,177)**	
One	2233 (20%)
Two	4344 (38.9%)
Three	3293 (29.5%)
Four and above	1307 (11.7%)
**Antenatal care visits**	
None	8530 (76.3%)
≥ 1 visits	2647 (23.7%)
**Maternal health status during pregnancy**	
None	11,081 (99.1%)
Known diabetic	96 (0.9%)
**Mode of delivery**	
Vaginal	9970 (89.2%)
CS	1207 (10.8%)
**Mothers who took multivitamin and foliate during pregnancy (n = 1342)**	
None	3 (0.2%)
Took multivitamin/folate	1339 (99.8%)

### Newborn baby characteristics

Of newborn babies the birth weight of 2322 (20.8%) newborn babies was < 2.5 kg.; 596 (5.3%) of the babies were twins; and 6901 (61.7%) were female. Babies alive and died/still births were 10,087 (90.2%) and 1089 (9.8%), respectively (Table 3).

**Table 3:** Characteristics of newborn babies born at Felege-Hiwot Comprehensive Specialized Referral hospital, from Sept. 2009 to Aug. 2014, Bahir Dar, Ethiopia.

Infant characteristics	Frequency (%)
**Child sex (n = 11,177)**	
Male	4276 (38.3%)
Female	6901 (61.7%)
**Birth weight**	
< 2.5 kg	2322 (20.8%)
≥ 2.5 kg	8855 (79.2%)
**Infant birth status**	
Alive	10,087 (90.2%)
Died/still birth	1090 (9.8%)
**Pregnancy status**	
Singleton	10,581 (94.7%)
Twins	596 (5.3%)
**Birth order**	
One	3045 (27.2%)
Two	4813 (43.1%)
Three	2502 (22.4%)
Four and above	817 (7.3%)

### The proportion of CAs among newborns

Out of the 11,177 participants, 69 had CAs. Thus, the observed proportion of CAs was 0.62% of which neural defects (46.4%), followed by unspecified congenital anomalies (20.3%), and cleft lip/palate (2.9%). Of the neural tube defects, anencephaly and spina bifida accounted for (24.64%), (21.74%), respectively and hydrocephalus (primarily secondary to spina bifida, but some may have hydrocephalus without spina bifida) was 19 (27.54%). CAs found registered as congenital malformations and defects and anomalies were considered as unspecified congenital anomalies (Table 4).

**Table 4:** The magnitude of congenital anomalies among newborn babies delivered at Felege-Hiwot Comprehensive Specialized Referral hospital, from Sept. 2009 to Aug. 2014, Bahir Dar, Ethiopia.

Types of congenital anomalies	Frequency (%)
**Neural tube defects (32 (46.4%))**	
Anencephaly	17 (24.64%)
Spina bifida	15 (21.74%)
Unspecified congenital anomalies	14 (20.3%)
Cleft lip/palate	2 (2.9%)
Club feet	2 (2.9%)
Total	69 (100%)

### Factors associate with CAs

Maternal factors significantly associated with CAs included urban residence adjusted odds ratio (AOR = 2.16; CI 1.33–3.51; p < 0.01) and lack of ANC visits (AOR = 0.24; CI 0.14–0.39; p < 0.001), whereas, such baby factors included female sex (AOR = 0.59; CI 0.37–0.95; p < 0.05) and less than 2.5 kg birth weight (AOR = 4.56; CI 2.76–7.55; p < 0.001) (Table 5).

**Table 5:** Factors associated with congenital anomalies, Felege-Hiwot Comprehensive Specialized Referral Hospital, Bahir Dar, Sept. 2009 to Aug. 2014.

Variables	Congenital anomaly		Odds ratio	
	Yes	No	Crude (95%CI)	Adjusted (95% CI)
**Mothers age (n = 11,177)**				
15–24 years	23 (1%)	2186 (99%)	1	1
25–34 years	38 (0.5%)	8003 (99.5%)	0.45 (0.26, 0.76)*	0.56 (0.32, 0.96)
≥ 35 years	8 (0.9%)	919 (99.1%)	0.83 (0.86, 1.86)	0.53 (0.22, 1.26)
**Residency**				
Urban	30 (1.0%)	2935 (99.0%)	2.14 (1.32, 3.45)**	2.16 (1.33, 3.51)**
Rural	39 (0.5%)	8173 (99.5%)	1	1
**Child sex**				
Male	36 (0.8%)	4240 (99.2%)	1	1
Female	33 (0.5%)	6868 (99.5%)	0.56 (0.35, 0.90)*	0.59 (0.37, 0.95)*
**Birth weight**				
< 2.5 kg	38 (1.6%)	2284 (98.4%)	4.74 (2.94, 7.62)***	4.56 (2.76, 7.55)***
≥ 2.5 kg	31 (0.4%)	8824 (99.6%)	1	1
**Mode of delivery**				
Vaginal	57 (0.6%)	9913 (99.4%)	1	1
C-section	12 (1.0%)	1195 (99.0%)	1.74 (0.93, 3.26)*	2.20 (1.16, 4.19)*
**Pregnancy status**				
Singleton	61 (0.6%)	10,520 (99.4%)	1	1
Twins	8 (1.3%)	588 (98.7%)	2.34 (1.12, 4.92)*	1.36 (0.64, 2.92)
**Antenatal care visits**				
No visit	30 (0.4%)	8500 (99.6%)	0.24 (0.14, 0.38)***	0.24 (0.14, 0.39)***
≥ 1visit	39 (1.5%)	2608 (98.5%)	1	1

Statistical significance at: *p-value < 0.05; **p-value < 0.01; ***p-value < 0.001.

## Discussion

This study revealed the proportion of CAs and associated factors among new babies delivered at Felege-Hiwot Comprehensive Specialized Referral hospital, Bahir Dar, Ethiopia. The aim of the study was to assess the situation and the causes of CAs among babies born at the hospital and to provide recommendations to concerned bodies to take preventive measures. The proportion of CAs noted in this study was 0.62%, lower than the findings of studies conducted in Addis Ababa, Ethiopia^23^, the University of Gondar comprehensive specialized hospital, Ethiopia^24^ and Addis Ababa and the Amhara region, Ethiopia^1^. Since both studies used record reviews, the difference in the proportion of CAs might be due to poor recording systems of the health facilities. In addition, the low proportion of CAs seen in the present study might be due to health care providers’ failure to record babies with CAs or perhaps due to negligence/carelessness or low ability of birth attendants to diagnose CAs. Moreover, the difference in frequency may be due to the fact that this study was conducted in one hospital just like the study conducted at the University of Gondar, while the studies conducted in Addis Ababa and the Amhara region, Ethiopia were done in many health facilities. The finding of current study was higher than that of Nigeria (0.4%)^25^. The justification for the differences might be methodological because studies vary by being facility or population based or retrospective or prospective. In addition, this difference might be due to environmental and genetic factors, diagnosing capability of health care providers and geographical settings. Again, this difference in the proportion of CAs could also be due to no use or use of folic acid/multivitamin during early pregnancy by one of the groups of participants.

Furthermore, our finding was the lowest of the studies conducted in East Africa^26^ and a facility based retrospective cross-sectional study in Nigeria^27^, which reported 2.5 and 2.8% of prevalence, respectively. These variations were due to the fact that the studies were influenced by bias or incomplete data because the methodology they used were retrospective cross-sectional. Moreover, these variations in the frequency were perhaps due to poor health facility recording ability/experience.

As has been observed in this study, maternal residence, environmental factors, sex of baby, birth weight, Caesarean-section (C-section) delivery, antenatal care visits, and maternal age had significant associations with CAs. From these findings we can learn that CAs are major problems of the community and needs the attention of the Ministry of Health, regional health bureau, zonal health departments and policy makers.

In this study, hydrocephalus was the most frequently observed anomaly followed by anencephaly and spina bifida. According to WHO report, neural tube defects (hydrocephalus, anencephaly and spina bifida) were the most prevalent anomalies worldwide^28^. Besides, a study conducted in Tanzania^20^ reported that central nervous system (CNS) defects commonly affect the organ system. In contrast, a study in Uganda^29^ pointed out that CNS defects did not commonly affect the organ system. This difference may be due to genetic and environmental factors.

In this study, maternal residence had a significant association with CAs which vary from place to place or with geographical locations; Urban resident mothers were 2.16 times more likely to have infants with CAs than rural dwellers^3^. This may be due to environmental factors, such as air pollution, radiation, exposure to chemicals and/or to pesticides.

In this study, more male babies (52%) were affected by CAs than female ones (48%). In contrast, a study conducted in Tanzania indicated that females were more affected by CAs than males^20^. However, a study done in Egypt reported that males were more likely to be affected than females^30^. This study findings were in line with findings of the present study.

In our study, only 20.8% of the babies free from CAs had < 2.5 kg birth weight, while 0.4% of babies had CAs. On the other hand, a study done in Egypt reported that about 71% of the infants had CAs^30^. In the present study, infants with < 2.5 kg birth weight were 4.56 times more likely to have CAs. A facility-based study conducted in Mekele, Ethiopia found that low birth weight was a significant contributing factor to CAs^31^. In contrast, a retrospective study done in Tanzania found that ≥ 2.5 kg birth weight^14^ was a significant contributing factor to CAs. That might be due to methodological differences or perhaps because the secondary data used was incomplete or due to maternal and fetal health conditions.

In the present study, 10.8% of the mothers had caesarean-section (C-Section). In another study conducted in Egypt 23.1% of the mothers had C-section, Breech presentations were noted in three-fold of the babies with CAs^30^. In this study, C-sections were 2.20 times more likely to be performed on babies with CAs.

Antenatal care was another significant contributing factor to CAs to which a negative relation was observed in this study, but many studies including WHO reports stated that it was vital for the prevention of some types of CAs^4,20,28^. Similarly, a study carried out in Nigeria showed that all mothers who had babies with CAs had ANC visit^27^. Again, another study reported that 67.7% of mothers who had babies with CAs made no ANC visits^21^. The possible explanation for this negative association might be that all antenatal care records were not documented by health care providers.

In this study, maternal advanced age was not associated with CAs. This finding was in line with that a study conducted in Addis Ababa and the Amhara region, Ethiopia^32^. Basically, the present study showed that 9.8% of babies were stillbirths/died due to adverse birth outcomes, which were not specified. The adverse birth outcomes could be due to CAs. Therefore, birth attendants must specify the causes of death of every newborn in the charts/logbooks. In addition, the Ministry of Health and regional health bureau should give training to birth attendants on CAs and ways of recording data. Lastly, we suggest that the interpretations of this finding should consider the number of adverse birth outcomes (deaths/stillbirths).

The limitation of the present study was that the data used were secondary which makes it difficult to generalize the findings in relation to the general population. The other limitation was that the cause and effect relationship could not be developed.

## Conclusion

This study revealed that neural tube defects (hydrocephalus, anencephaly and spina bifida) were the commonest anomalies in the study area and require due attentions. Low birth weight, baby sex, no ANC visits and urban residence were factors related with CAs. In addition, babies with CAs were more likely to be born through C-section. This study noted that many newborn babies were died/stillbirth due to unspecified causes. This suggests that the chance of death of newborn babies with CAs could be higher than that of babies born alive. Therefore, we recommend that the Ministry of Health, concerned bodies, policy makers and the Government of the country should give due attention to CA cases.

## Data Availability

The data set supporting this study are available in the manuscript.

## Abbreviations

CA: Congenital anomaly
CAs: Congenital anomalies
CI: Confidence interval
ANC: Antenatal care
MPH: Master of public health
WHO: World Health Organization
MRI: Magnetic resonance imaging
CT scan: Medical examination by using computer to produce inside body image
OR: Odds ratio
